# Incidence and Prevalence of Early-Onset Dementia in Finland

**DOI:** 10.1212/WNL.0000000000209654

**Published:** 2024-07-24

**Authors:** Johanna Krüger, Mikko Aaltonen, Kalle Aho, Sami Heikkinen, Ave Kivisild, Adolfina Lehtonen, Laura Leppänen, Iina Rinnankoski, Helmi Soppela, Laura Tervonen, Noora-Maria Suhonen, Annakaisa Haapasalo, Anne M. Portaankorva, Anna Mäki-Petäjä-Leinonen, Päivi Hartikainen, Kasper Katisko, Eino Solje

**Affiliations:** From the Research Unit of Clinical Medicine (J.K., A.L., L.L., I.R., L.T., N.-M.S.), Neurology, University of Oulu; MRC (J.K., L.T., N.-M.S.); Neurocenter (J.K., L.T., N.-M.S.), Neurology, Oulu University Hospital; Law School (M.A., A.M.-P.-L.), University of Eastern Finland, Joensuu; Institute of Clinical Medicine - Neurology (K.A., S.H., A.K., H.S., K.K., E.S.); A.I. Virtanen Institute for Molecular Sciences (A.H.), University of Eastern Finland, Kuopio; Clinical Neurosciences (A.M.P.), Faculty of Medicine, University of Helsinki; and Neuro Center - Neurology (P.H., E.S.), Kuopio University Hospital, Finland.

## Abstract

**Objectives:**

Current epidemiologic data of early-onset dementia (EOD), characterized by the onset of the disease before the age of 65, are notably scarce.

**Methods:**

We evaluated the incidence (from January 2010 to December 2021) and prevalence (on December 31, 2021) of EOD and its subtypes in 2 defined areas in Finland. All visits at the dementia outpatient clinics were manually retrospectively reviewed and reassessed (N = 12,490).

**Results:**

In the population aged ≤65 years, crude incidence of EOD was 12.3/100,000 persons at risk/year based on 794 new cases from January 1, 2010, to December 31, 2021. Incidence rates for EOD were 20.5 and 33.7 per 100,000 person years in the age group of 30–64 and 45–64 years, respectively. The prevalence of EOD was 110.4 in the age group of 30–64 years and 190.3 in the age group 45–64. Alzheimer disease (AD) (48.2%) and behavioral variant frontotemporal dementia (12.7%) were the most frequent subtypes. The incidence of AD increased during the follow-up, whereas incidence of other forms of EOD remained stable.

**Discussion:**

We found higher incidence rates of EOD than previously reported. Unlike other forms of EOD, the incidence of early-onset AD seems to be increasing.

## Introduction

The growing prevalence of dementia presents substantial challenges for public health and economy, with estimated costs soaring to 1,313.4 billion US dollars globally.^1^ Approximately 10% of dementia is diagnosed at young age (≤65 years), categorized as early-onset dementia (EOD).^2,3^

In the era of emerging disease-modifying drugs for Alzheimer disease (AD)^4^ and other forms of dementia, access to up-to-date epidemiologic data for these disorders becomes increasingly crucial. Effective health policy planning and the design of new drug trials for neurodegenerative diseases rely on accurate and updated real-world epidemiologic data.

The existing epidemiologic data of EOD^2,5,6^ are of limited applicability due to small sample size or nonavailable reviews of clinical diagnoses, that is, diagnoses often rely on single–time-point register collections. Furthermore, register-based analyses are difficult to validate considering diagnostic accuracy or whether the initially diagnosed disease correctly represents the natural progression of the condition.

This study provides up-to-date valid numbers for the incidence and prevalence of EOD. We systematically collected all EOD clinical diagnoses from 2 university hospital districts in the defined areas of Finland.

## Methods

### Standard Protocol Approvals, Registrations, and Patient Consents

This study represents a noninterventional registry study, and no additional data were collected directly from the study individuals. The Finnish Law (552/2019) does not mandate consent for retrospective studies without patient contact, so no Ethical Committee evaluation was needed. Finnish Social and Health Data Permit Authority Findata approved the research protocol (THL/2841/14.02.00/2022). This study is part of DEGE-RWD, research project (NCT06209515), coordinated by Neurocenter Finland.

### Study Cohorts and Clinical Review

We conducted an epidemiologic study in the provinces of Northern Ostrobothnia (37,149 km^2^) located in Northern Finland and Northern Savonia (20,367 km^2^) in Eastern Finland in the 30–64-year-old inhabitant populations of 174,249 and 106,579, respectively, on December 31, 2021. Kuopio University Hospital (KUH) and Oulu University Hospital (OUH) represent tertiary level neurologic clinics in these areas and are the primary regional referral centers for all citizens ≤65 with neurodegenerative disease-related cognitive complaints. Thus, the EOD diagnostics are performed exclusively within these 2 centers. All patients admitted to KUH and OUH Neurology outpatient clinics with a progressive neurodegenerative disease during 2010–2021 were identified from patient data registries (N = 12,490). For inclusion and exclusion criteria, see eMethods.

### Statistical Analyses

Incidence of EOD was calculated by dividing the total number of new EOD cases in 2010–2021 by the number of person-years in the 2 regions during the same period. The prevalence of EOD was calculated by using population statistics at the end of year 2021 as the denominator, whereas all individuals with a diagnosis of EOD alive and aged ≤65 on December 31, 2021, were included in the numerator. For more detail on statistical analyses, see Supplementary File (eMethods).

## Results

In total, patient charts of 12,490 individuals (8,386 from KUH and 4,104 from OUH) were initially reviewed. Of these, N = 794 patients (458 from OUH and 336 from KUH) were included into this study as individuals fulfilling the criteria for progressive EOD. The most common clinical diagnosis was AD (48.2%), followed by behavioral variant of frontotemporal dementia (bvFTD) (12.7%) and vascular cognitive disorders (12.2%). The total study sample with eventual diagnoses is described in Table 1. Of the patients with AD, 62.2% had CSF amyloid and tau biomarkers measured, indicating AD pathology.

**Table 1:** Primary Diagnoses in the Sample (n = 794)

Diagnosis	N	%	M/F
Alzheimer disease continuum	421	53	175/246
Alzheimer disease	383	48	157/226
Posterior cortical atrophy	30	4	17/13
Frontal Alzheimer disease	8	1	^a^
Frontotemporal dementia spectrum	180	23	96/84
Behavioral variant of frontotemporal dementia	101	13	48/53
Nonfluent variant primary progressive aphasia	17	2	10/7
Semantic variant primary progressive aphasia	7	1	4/3
Logopenic variant primary progressive aphasia	^a^	^a^	^a^
Progressive supranuclear palsy	21	3	13/8
Corticobasal degeneration	8	1	4/4
Frontotemporal dementia with amyotrophic lateral sclerosis (FTD-ALS)	25	3	16/9
α-Synucleopathies	46	6	33/13
Dementia with Lewy bodies	31	4	24/7
Multiple system atrophy	15	2	9/6
Other	147	19	89/58
Vascular cognitive disorders	97	12	59/38
Alzheimer disease and dementia with Lewy bodies	8	1	^a^
Alzheimer disease with vascular dementia	22	3	16/6
Huntington disease	15	2	6/9
Early-onset dementia, undefined	5	1	^a^

Abbreviations: F = female; M = male.

a The exact number of individuals is not displayed if it is fewer than 3 people to ensure the privacy of individuals.

**Figure 1:**
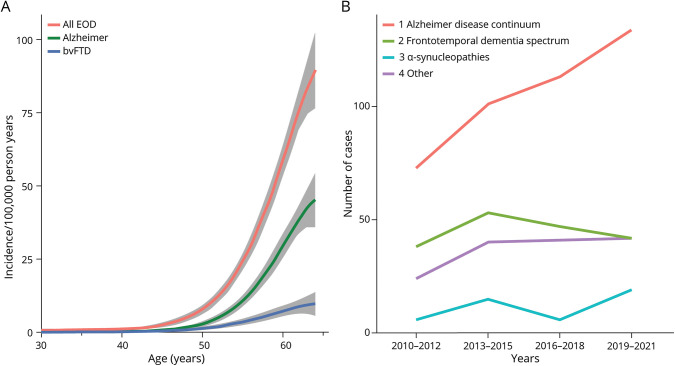
Predicted Incidence by Age and New Early-Onset Dementia Cases by Diagnosis Group, 2021–2021 (A) Predicted* incidence (/100,000 person years) by age. Any early-onset dementia (EOD), Alzheimer disease (AD), and behavioral variant of frontotemporal dementia (bvFTD). * Age-specific predicted incidence and 95% confidence intervals calculated from Poisson regression models with cubic age polynomial. (B) New EOD cases by diagnosis group in 2010–2021.

Based on 794 new cases between January 1, 2010, and December 31, 2021, the crude incidence of EOD was 12.3/100,000 persons at risk/year in the population aged 0–65. The incidence for persons at risk was 20.5/100,000 and 33.7/100,000 in the populations aged 30–64 years and 45–64 years, respectively. The incidences for different age groups and sexes are presented in Table 2. In the population aged 30–64 years, the European Standard Population age-standardized incidence rate was 17.8/100,000 and the World Standard Population adjusted incidence rate was 15.1/100,000. During the study period of 12 years, the incidence of early-onset AD increased progressively, while the incidence rates of other forms of EOD remained relatively stable (Figure 1).

**Table 2:** Age-Specific and Sex-Specific Incidence in 2010–2021 and Prevalence of Early-Onset Dementia at the End of the Year 2021 in Finland (Northern Ostrobothnia and Northern Savonia Districts)

Incidence									
Age range	Person years			All		Male		Female	
	All	Male	Female	n	Rate	n	Rate	n	Rate
30–34	481,310	252,184	229,126	^a^	^a^	^a^	^a^	^a^	^a^
35–39	471,775	245,730	226,045	4	0.8	^a^	^a^	^a^	^a^
40–44	452,567	234,754	217,813	12	2.7	9	3.8	3	1.4
45–49	469,029	241,333	227,696	16	3.4	9	3.7	7	3.1
50–54	511,088	259,904	251,184	60	11.7	36	13.9	24	9.6
55–59	538,193	271,847	266,346	218	40.5	107	39.4	111	41.7
60–64	541,706	272,305	269,401	400	73.8	202	74.2	198	73.5
30–64	3,465,668	1,778,057	1,687,611	711	20.5	366	20.6	345	20.4
45–64	2,060,016	1,045,389	1,014,627	694	33.7	354	33.9	340	33.5

Prevalence									
Age range	Persons			All		Male		Female	
	All	Male	Female	n	Prev	n	Prev	n	Prev
30–34	40,059	21,060	18,999	^a^	^a^	^a^	^a^	^a^	^a^
35–39	41,432	21,593	19,839	^a^	^a^	^a^	^a^	^a^	^a^
40–44	39,617	20,484	19,133	3	7.6	^a^	^a^	^a^	^a^
45–49	36,512	18,987	17,525	7	19.2	6	31.6	^a^	^a^
50–54	37,789	19,271	18,518	18	47.6	11	57.1	7	37.8
55–59	42,117	21,318	20,799	74	175.7	43	201.7	31	149.0
60–64	43,302	21,425	21,877	205	473.4	105	490.1	100	457.1
30–64	280,828	144,138	136,690	310	110.4	169	117.2	141	103.2
45–64	159,720	81,001	78,719	304	190.3	165	203.7	139	176.6

Abbreviation: Prev = prevalence.

No statistical significance between male and female patients.

a The exact number of individuals is not displayed if it is fewer than 3 people to ensure the privacy of individuals.

We identified altogether 310 patients aged ≤65 with EOD on census date of 31 December 2021. The prevalences of EOD were 110.4 and 190.3 per 100,000 population in the age group of 30–64 years and 45–64 years, respectively. In the age group of 30–64 years, prevalence was 96.9 per 100,000 when age-adjusted for European Standard Population and 82.2 when age-adjusted for World Standard Population.

The prevalences for different age groups for male and female patients are presented in Table 2. No statistical significance was seen between sexes. The incidence and prevalence numbers for distinct clinical phenotypes of EOD are presented in eTable1.

## Discussion

In this study, we report higher EOD incidence rates compared with studies from Italy, Spain, and Norway.^7-9^ Notably, our World Standard Population age-adjusted incidence rate (15.1/100,000 for 30–64-year population) and the European Standard Population age-adjusted incidence rate (17.8/100,000 for the population aged 30–64 years) were also higher than the rates calculated in a recent meta-analysis for the same age group (11/100,000 and 14.4/100,000, respectively).^5^ The higher incidence rates in our study may reflect the study methodology, which enabled us to detect close to all EOD cases from the study areas. Of interest, the incidence rates of early-onset AD increased steadily, while the incidence figures of other forms of EOD remained relatively stable during the study period. Since the diagnoses were carefully reviewed and classified to diagnostic groups by using uniform criteria, and the incidence numbers for other causes of dementia did not increase, our results may indicate real increase of early-onset AD. However, it might be partly due to the advancements in the awareness or diagnostics, for instance the increased use of CSF biomarkers.

The crude prevalence rates were higher in our study compared with the Norwegian study.^10^ When comparing age-adjusted numbers, our World Standard Population adjusted prevalence for the population aged 30–64 years (82.2/100,000) was lower than the rate reported in a large meta-analysis (119.0/100,000).^6^ However, most of the studies in this meta-analysis reported prevalence of EOD only in the age group of 40–65 years, while only a few studies had evaluated the prevalence in younger age groups.

As strengths, we consider the 12-year duration of data collection (encompassing substantial 6,467,028 person-years of the referenced population) and the health care system structure in Finland resulting in the 2 university hospitals serving as centers for all suspected patients with EOD. Furthermore, every diagnosis was thoroughly reassessed, and all the health care visits following the initial diagnosis were taken into account. Moreover, public hospitals in Finland are known for their high quality in diagnosing EOD, including MRI and/or PET imaging, usually CSF analysis of AD biomarkers, and comprehensive neuropsychological assessments.

As limitations, some cases might have been missed because of loss of insight and thus patient not being referred for diagnostic evaluations. However, this is unlikely due to the regular health checks at occupational health care or mandatory health checks for unemployed people. Furthermore, the social security system of Finland mandates medical diagnosis to justify societal benefits, such as pensions.

In conclusion, our findings suggest higher overall EOD incidence than previously reported, and steadily increasing incidence rate of specifically AD during the study period.

## Appendix Authors

**Table:**

Name	Location	Contribution
Johanna Krüger, MD, PhD	Research Unit of Clinical Medicine, Neurology, University of Oulu; MRC; Neurocenter, Neurology, Oulu University Hospital, Oulu, Finland	Drafting/revision of the manuscript for content, including medical writing for content; major role in the acquisition of data; study concept or design; analysis or interpretation of data
Mikko Aaltonen, PhD	Law School, University of Eastern Finland, Joensuu	Drafting/revision of the manuscript for content, including medical writing for content; major role in the acquisition of data; study concept or design; analysis or interpretation of data
Kalle Aho, BM	Institute of Clinical Medicine - Neurology, University of Eastern Finland, Kuopio	Drafting/revision of the manuscript for content, including medical writing for content; major role in the acquisition of data
Sami Heikkinen, MD	Institute of Clinical Medicine - Neurology, University of Eastern Finland, Kuopio	Drafting/revision of the manuscript for content, including medical writing for content; major role in the acquisition of data
Ave Kivisild, MD	Institute of Clinical Medicine - Neurology, University of Eastern Finland, Kuopio	Drafting/revision of the manuscript for content, including medical writing for content; major role in the acquisition of data
Adolfina Lehtonen, BM	Research Unit of Clinical Medicine, Neurology, University of Oulu, Finland	Drafting/revision of the manuscript for content, including medical writing for content; major role in the acquisition of data
Laura Leppänen, BM	Research Unit of Clinical Medicine, Neurology, University of Oulu, Finland	Drafting/revision of the manuscript for content, including medical writing for content; major role in the acquisition of data
Iina Rinnankoski, BM	Research Unit of Clinical Medicine, Neurology, University of Oulu, Finland	Drafting/revision of the manuscript for content, including medical writing for content; major role in the acquisition of data
Helmi Soppela, BM	Institute of Clinical Medicine - Neurology, University of Eastern Finland, Kuopio	Drafting/revision of the manuscript for content, including medical writing for content; major role in the acquisition of data
Laura Tervonen, MD	Research Unit of Clinical Medicine, Neurology, University of Oulu; MRC; Neurocenter, Neurology, Oulu University Hospital, Oulu, Finland	Drafting/revision of the manuscript for content, including medical writing for content; major role in the acquisition of data
Noora-Maria Suhonen, PhD	Research Unit of Clinical Medicine, Neurology, University of Oulu; MRC; Neurocenter, Neurology, Oulu University Hospital, Oulu, Finland	Drafting/revision of the manuscript for content, including medical writing for content; major role in the acquisition of data; study concept or design
Annakaisa Haapasalo, PhD	A.I. Virtanen Institute for Molecular Sciences, University of Eastern Finland, Kuopio	Drafting/revision of the manuscript for content, including medical writing for content; study concept or design; analysis or interpretation of data
Anne M. Portaankorva, MD, PhD	Clinical Neurosciences, Faculty of Medicine, University of Helsinki, Finland	Drafting/revision of the manuscript for content, including medical writing for content; study concept or design; analysis or interpretation of data
Anna Mäki-Petäjä-Leinonen, LL.D.	Law School, University of Eastern Finland, Joensuu	Drafting/revision of the manuscript for content, including medical writing for content; study concept or design; analysis or interpretation of data
Päivi Hartikainen, MD, PhD	Neuro Center - Neurology, Kuopio University Hospital, Finland	Drafting/revision of the manuscript for content, including medical writing for content; study concept or design; analysis or interpretation of data
Kasper Katisko, MD, PhD	Institute of Clinical Medicine - Neurology, University of Eastern Finland, Kuopio	Drafting/revision of the manuscript for content, including medical writing for content; study concept or design; analysis or interpretation of data
Eino Solje, MD, PhD	Neuro Center - Neurology, Kuopio University Hospital; Institute of Clinical Medicine - Neurology, University of Eastern Finland, Kuopio, Finland	Drafting/revision of the manuscript for content, including medical writing for content; major role in the acquisition of data; study concept or design; analysis or interpretation of data
